# Memoir Intended to Show That the Cause of the Entry and Motion of the Sap in Plants, Ought to Be Attributed to Vacuities Created in Their Vessels by Transpiration

**Published:** 1805-09-01

**Authors:** I. B. Bizet

**Affiliations:** Amiens


### Memoir intended to show that the Cause of the Entry and Motion of the Sap in Plants, ought to be attributed to Vacuities created in their Vessels by Transpiration. *By* I. B. Bizet, *of Amiens.*

[ Extracted from the Journal de Physique, tom. lix. p. 43. ]

It is known, and perhaps the knowledge would stand high in the calendar of human acquirements, if its triviality does not prevent its admission among them, that vegetables receive their nourishment, at least the most substantial part of it, such as their sap, by means of the roots; but it does not appear that we have yet attained to the discovery of the cause of its entrance into their interior. Animals have in themselves the faculty of choosing their food, and of conveying it into organs, where it is converted into their substance; the vegetable has no other faculty but that of receiving the juices it meets with in the spots of earth and water to which its roots extend. It is true that these parts of a vegetable are all disposed for the reception of such juices; they are covered with an infinity of mouths destined and ready to serve as entrances to the canals designed to distribute them through the whole of their interior. But what introduces them into these canals? What is the cause of their entrance into, and circulation in them?

Grew, a celebrated English philosopher, was of opinion that the sap must be greatly rarified, and in some measure reduced to vapour before it could enter into plants, and that it was only raised in their vessels through the levity it acquired in this state.

To this it was answered, by M. Duhamel Dumonceau, that sap was never found in plants, except in a liquid form.

If he had replied that this was because the vapours were condensed in their roots, it might have been asked, what refrigerants of sufficient activity could these vapours have encountered there, to be condensed so early as to be found only in a liquid state, while the roots necessarily participate in the same degree of temperature as that of the earth or water with which they are surrounded; a temperature which must equally have opposed the formation of these vapours?

It has been attempted to explain the cause of the introduction and motion of the sap in plants by the dilatation and and condensation of the air they contain. It has been said, that when the air included in the trachea of their roots become rarefied, it pressed upon the vessels filled with the sap, and by this impulse forced it towards the upper part of the vessels; and that when the volume of air in these trachea was diminished by condensation, a vacuum was formed in the sap vessels of these parts, which produced a suction, and attracted new sap thither.

But the air in the trachea of the roots of plants can only be dilated by heat; now when the heat dilated this air, it must also have dilated, at the same time, and more powerfully, that of the trachea of all its other parts, since they are more exposed to it; the impulse of the pressure must therefore be stronger towards the inferior than towards the superior parts of vegetables. The dilatation of the air in plants, produced by this cause, would consequently be more disposed to drive the sap from the upper parts of them, and to cause it to pass downwards to the roots, than to occasion such vacuities in them as would produce suction.

Others have been satisfied to assimilate the first entrance of the sap into plants, to that of water into spongy bodies or into capillary tubes, where nevertheless they could only, by either of these methods, cause it to ascend some fractions of a metre; and still less could they thus convey the sap to the height of the cedars of Lebanon, or even to that of the oaks of our forests.

The inadequacy of these causes assigned for the entrance and motion of the sap in plants, has occasioned other observers to be contented to attribute them to heat, or, which is the same thing, to the influence of the sun. It is true that while a plant vegetates, the sap is always most abundant in the warmest weather; but it appears to me that to attribute its entrance and its motions to this cause, is to assign the general cause of vegetation, or the universal cause of all its phenomena, to an individual phenomenon; and as each of these, in addition to the general, must have a peculiar cause, the motion of the sap must also have the same, the discovery of which is the object of the present memoir. Besides, how is it possible to suppose that heat is the particular cause of the entry of the sap, when it is this which occasions it to exude so abundantly in transpiration? Effects so opposite imply different causes. It may also be asked of those who support such an opinion, how heat can introduce these nutritive juices into into such plants as push their roots to a depth in the earth at which the influence of the sun is never sensible.

Hence M. Duhamel, enlightened by the knowledge of the philosophers who had preceded him, and assisted by that of his contemporaries, after having weighed their opinions, repeated and examined their experiments, extended their researches, and greatly multiplied his own, was compelled to acknowledge, that *all he had said on the cause which determined the ascent of the sap in plants, must only be considered as simple conjectures.* These are his own words in his Physique des Arbres, tom. ii. p. 264.

But if M. Duhamel could only offer conjectures on the cause of the motion of sap in plants, can I flatter myself with having discovered its true cause? I am, at least, authorized to believe so, since the theory on which I rest my proofs, is established on facts, and these facts are confirmed by numerous and constant observations, which leave no room for the smallest doubt.

It is known, as I have already observed, that the juices which nourish plants are introduced into them by their roots; it is also known that these juices rise to the extremities of their stems and their branches, whence it is probable that they pass between their internal parts and their bark, where they continually form new layers, which progressively increase the bulk of them, and consequently that of the roots, to which they descend, and also reach to their extremities.

The nature of the vessel which conduct these juices in plants is not yet known; it has only been discovered from multiplied experiments, that such of these vessels as receive them at their entrance into the roots, follow their fibrous or ligneous parts through all their extent. Are these vessels of a nature to be compared to the arteries and veins of animals; or, which is less probable, are they only cellular or spongy textures, which by communicating with each other, distribute the juices into all the parts of the plant? On this subject there is nothing but uncertainty; but it may be considered as an established fact, that those conductors of sap in vegetables, whatsoever they may be, retain it in such a manner that no part of it can escape except by transpiration, and only by the passages destined for that purpose; and that what does escape is only the constituent parts of the sap; the lymphatic parts reduced to vapour; the oxygen, hydrogen, and azotic gases, which plants continually emit into the atmosphere; but it is never sap. A proof of this is, that the extravasation of sap is a mortal disease to plants, and always, or nearly always, at least while they vegetate, is the cause of a state of debility and languor. Hence arises the maxim in gardening, which directs the covering and taking care of the wounds made in trees when they are pruned, as well as those arising from any other cause.

If the sap cannot escape from the vessels which distribute it through the plant, these may for that reason be compared to tubes as far as regards the sap, and their properties in this respect are the same; now, it is universally known that if a vacuum is made in a tube, the end of which is plunged into a liquid, the vacuum attracts it, and causes it to ascend in the tube. I shall not, in describing the cause of this phenomenon, use the language of the learned of former days, and say that it is because *Nature abhors a vacuum.* Torricelli and Pascal opened the paths which led to the discovery of this cause, by enabling us to discover the properties of a vacuum. The same cause produces the same effect in Torricelli's tubes, and in the vessels of plants; it is also this cause which is the principle of the power of suction, which M. Duhamel and several of his contemporaries suspected in their roots. To arrive at this cause, it would only have been necessary for them to have discovered that of the suction, which is also the subject of this inquiry.

To perceive the reality, and all the efficacy of this cause, it is sufficient to consider a vegetating plant as being what in reality it is, a compound of vessels, all the capacity of which is filled with the juices or sap it contains, and the lower extremities of which are enveloped in humid earth, from which it draws its nourishment. Were it necessary to bring proofs of this assertion, they would be readily found in the state of humidity and verdure of all its parts.

These vessels are successively emptied, more or less, of their juices by the effects of the transpiration they experience, which necessarily creates vacuities in them, and these produce the same effects in the vessels as those occasioned by the piston in the Torricellian tubes, or in air pumps; that is to say, they attract the juices from the lower parts of the vessels until they are filled.

The parts of the vegetable in which these vacua are first formed, are those most exposed to heat or to the action of the sun. These exposed parts, by gradually attracting the sap from those less exposed, give rise to similar vacua there, which, in their turn, act on the sap of the roots, and and consequently produce that power of suction suspected by authors; this attracts the water, or humidity of the earth, which, by this mechanism, becomes the nourishment of the vegetable.

But Torricelli and Pascal have shown, that it was the weight of the atmosphere on the liquid which caused it to rise in the tubes in their experiments; now, it may be said, how can this weight reach the fluid which enters into the roots of vegetables, and press on them to force their entrance, while the roots are frequently at considerable depths in the earth, which, itself bears all the weight, and consequently relieves the liquid from it? Besides, it may be added, in the experiments of Torricelli and Pascal, the tubes made use of were of a capacity which offered no obstacle to the entrance and motion of the liquid they contained; and those which receive the sap in plants are very capillary tubes and almost imperceptible, in which the liquids introduced meet greater obstructions in proportion to the friction they experience. By what means can the power of the weight of the atmosphere, at least weakened at such depths, overcome them?

The weight of the atmosphere which bears on the surface of the earth and compresses it, also compresses all the waters it meets with there. One of the principal effects of this pressure on the waters is to prevent them from being carried off and dissipated in vapour; and this force of compression is so powerful that it raises a column of mercury equal in weight to a column of water thirty-two feet in height, as is shown by the Torricellian experiments; there is nothing in action except caloric, which has the power of reducing a part of these waters, proportionate to its intensity, to vapour, and carrying it into the region of clouds. But what remains in the earth does not cease to be compressed by the atmospheric weight; this power alone does and can cause it to enter and be dispersed in its bosom, since without that, the water, instead of descending and penetrating into it, would necessarily be elevated as in a vacuum.

The waters spread through the earth are therefore in a state of compression in it; they must, consequently, enter into the vacuities they meet with in the roots, and rise in the vessels of the plants, for the same reason as they ascend and fill the tubes in the experiments of Torricelli and Pascal.

We shall however presently see that this cause of the entrance and circulation of the sap in the vessels of plants is not absolutely necessary.

It is true that these vessels, as well as their entrances in the roots, offer only very capillary passages to the water or the sap; but, 1st, this circumstance is so far from being a hindrance to the introduction of it into the roots, that, on the contrary, it appears to be very favourable to it; it is known that water enters and rises in capillary tubes without a vacuum being necessary; the water or sap must therefore enter more readily into the roots of plants where there are vacuities which attract it.

2d. I do not mean to be understood that the sap meets with no obstacles to its ascent in plants, or that its motion in their capillary vessels are as free and as rapid as that of the water in the tubes in the experiments mentioned above; but these obstacles, without injuring the efficacy of its circulation, only render it slower, and serve to explain some phenomena of vegetation, which, otherwise, perhaps, it would be difficult to understand.

There are few who have not observed that in the great heats of summer, many plants, particularly those most exposed to the sun, the extremity of whose branches, and also other parts which have not acquired sufficient solidity to be supported by the strength of their fibres, become relaxed and hang almost perpendicularly towards the earth; this gives them an appearance of languor, which may be said to be almost momentary, for as soon as the sun has disappeared, these drooping plants regain the vigour they had before.

The cause of this phenomenon can only be sought in the great quantity of sap which these parts of the plants lose by transpiration, while they are heated by the sun; they were only supported before because they were filled and distended by the sap. The transpiration they afterwards experience occasions great vacuities in them, and the parts which were strengthened by being filled, cease to be so, which causes them to bend towards the earth, and they remain drooping because they are filled slowly, on account of the obstacles which the new sap meets with in the capillary vessels of the plants to stop or retard its passage from the roots.

Another objection may be offered, which at the first blush appears more plausible. It may be said, if the cause which occasions the elevation of the sap in plants be the same as that which makes the water ascend in tubes, in the experiments of Torricelli and Pascal, this can only raise raise the water in them to the height of thirty-two feet, how then can it carry the sap to the tops of the tallest trees, which are seventy or eighty feet and upwards?

This objection only appears plausible because we have not yet determined in a precise manner the cause which makes the water rise in the tubes in the above experiments. At first, this cause appears to be only the pressure of the atmosphere; this pressure, however, is evidently but a remote cause; the true, the immediate cause which occasions the ascent of the water in these experiments, must be sought in the vacua which are made in them; it is evident that the water only rises as they are made and in proportion to them. Torricelli and Pascal stood in need of the pressure of the atmosphere to be able to make a vacuum, and hence they were unable to produce them beyond a height of thirty-two feet; but the Ruler of Nature has created a profusion whi h are independant of this pressure, in the economy of vegetation.

